# Supplementary material for: Tracing Autism Traits in Large Multiplex Families to Identify Endophenotypes of the Broader Autism Phenotype
Source: Int J Mol Sci. 2020 Oct 27;21(21):7965. doi: 10.3390/ijms21217965 (PMC7663259; doi:10.3390/ijms21217965)
Supplement: Supplementary file 1 [file ijms-21-07965-s001.zip › Broader Autism Phenotype Interview v3.pdf]

The Broader Autism Phenotype Interview (BAPI)

Developed by the Collaborative Autism Study Team, The University of Melbourne
Version 3.0 (2020)

Administration time: 30 minutes to 1 hour

Instructions:
The BAPI is presented as a series of interview questions on personality and lifestyle. Before administration, emphasise that this is not a test, and that there are no right or wrong answers. It is important to ensure that participants feel relaxed and comfortable to encourage truthful responding, and to minimise self-enhancement or underreporting of associated Broader Autism Phenotype (BAP) traits. The manner and quality of response (e.g pragmatic language, intensity, degree of elaboration) can be highly informative, and should be carefully observed throughout the session.

Participants are rated on 33 BAP traits based on information gathered on the BAPI. For more details on scoring, please refer to Trevis et al (2020).

Please include the follow citation when using the BAPI:

Trevis, K. J., Brown, N. J., Green, C. G., Lockhart, P. J., Desai, T., Vick, T., Anderson, V., Pua, E. P. K., Bahlo, M., Delatycki, M. B., Scheffer, I. E., and Wilson, S. J. (2020). Tracing Autism Traits in Large Multiplex Families to Identify Endophenotypes of the Broader Autism Phenotype. *International Journal of Molecular Sciences.*

The Broader Autism Phenotype Interview (BAPI) Scoring Sheet

Participant: **Rated by:**

Date:

Cluster	Trait	Rating			
		0	1	2	3
**Aloof**<br>*Difficulties relating to others' emotions and expressing own emotions*<br>**____ / 27 =_____**<br>**Cutoff: >0.20**	Aloof personality style				
	Difficult or limited interpersonal relationships				
	Reduced emotional empathy				
	Limited capacity to develop rapport with assessors				
	Reduced affection				
	Awkward social interactions				
	Opinionated in conversation				
	Reduced cognitive empathy				
	Little appreciation of humour (During Cartoon task)				
**Pedantic**<br>*Self-focused and technical in interactions*<br>**____ / 33=_____**<br>**Cutoff: >0.14**	An unusual or awkward greeting style				
	Unusual eye gaze				
	Speech has little variation in tone (i.e. monotonous)				
	Unusual speech volume				
	Precise articulation and language				
	Terse pragmatic style				
	Overly technical language				
	Narcissistic personality style				
	Focus on technicalities or minutiae				
	Fastidious regarding personal appearance				
	Self-perception incongruent with views of others				
**Socially unaware**<br>*Poor self-regulation and reciprocity in conversation*<br>**____ / 21 =_____**<br>**Cutoff: >0.17**	Reduced capacity for clear narrative				
	Difficulty answering open ended questions				
	Making inappropriate or awkward comments either on history or during assessments				
	Tangential pragmatic style				
	Tendency to monologue rather than participate in reciprocal conversation				
	Tendency to anger easily				
	Reduced quantity of verbal output				
**Obsessive**<br>*Regimented approach to life and tendency to ruminate*<br>**_____ / 18=_____**<br>**Cutoff: >0.25**	Hobby/interest of unusual intensity, or restricted in range of interests relative to peers				
	Large collections or hoarding of items				
	Fastidious cleaning				
	Preference for structure to activities of daily living				
	Recurrent thoughts that are not distressing				
	Excessive worry				

0=None; 1=Mild; 2=Moderate; 3=Severe **Total: /99**

The Broader Autism Phenotype Interview (BAPI)

I will ask you some questions about how you normally interact with other people and some questions about your personality and lifestyle. Just answer as best you can and try to think about how you have been most of your life rather than about how you may have behaved in certain special times of your life. There are no right or wrong answers. All of the questions ask about personality features that are found in everyone but vary in degree from person to person.

A good place to start might be for you to tell me a little bit about yourself?
How would you describe yourself?
What are you like as a person?

Repetitive or Stereotyped Behaviours or Interests

A **Can you tell me about some things you enjoy doing?**
What do you do in your spare time?
Do you have any hobbies, special interests or collections?
How much time do you spend on your hobbies/activities?

Do you feel that your hobby/activities prevents you from doing other things that you want to do or that you should do?
What things does it prevent you from doing?

Does this ever interfere with your work or prevent you from spending time with others?

Have other people ever said that you spend too much time on your hobby?

Do you ever feel a strong need to engage in your hobby?

Do you ever get so absorbed in your hobby that you lose track of what is going on around you?
Does it happen rarely or often?

Does it bother you if someone talks to you or distracts you from your hobby?
Do you sometimes feel bothered or often feel bothered?
Does this ever cause problems in your social life or relationships?
[EXAMPLE] Do you ever have arguments with someone about your hobby?

Scoring: __________

0 They may rarely have a strong urge to engage in their hobby. The hobby does not negatively impact on other aspects of their life and they are not often absorbed in their hobby and do not mind being interrupted when engaging in their hobby.
1a Spends a lot of time on a hobby (good proportion of their spare time). This interferes with other aspects of life (e.g. planned grocery run) without causing any substantial difficulties. May or may not feel an urge and be absorbed when engaging in their hobby
1b They feel a need to engage in their hobby and become absorbed in their hobby. They are bothered by being disturbed when engaging with the hobby. The hobby may or may not interfere with minor aspects of life. They do not spend a lot of time on their hobby.
2a Spends a lot of time on a hobby (most of their spare time). The activity interferes with important aspects of life (work, social functioning).
2b They feel a need to engage in their hobby, they are highly focused when doing the hobby and are not happy when disturbed from their hobby which causes problems with social functioning.
2c Spends a lot of a time on a hobby and feel a need to engage in their hobby, and/or are highly focused when doing the hobby, and/or dislike being disturbed from their hobby.

B **What do you do on a typical day?**

Do you have a daily routine?
Can you tell me about your daily routine?

Does it bother you if you have to change your routine or your daily plans?

Why is that?
Does it bother you if you can't [insert] at the usual time?
Does it bother you if you have to change the order in which you do thiings?
Does it bother you if you have to cancel an activity or replace it with another one?

[If no indication of rigidity yet]

Is there anything that you like to do in a particular way or with a set routine?
Does it bother you if you have to do [insert] in a different way than usual?

* Look for typical responses such as a very brief description of breakfast, lunch, dinner, work, recreation

Scoring: __________

0 Will give a general overview of their routine. Flexible to changes in routine.
1a Somewhat detailed in their routine description but adaptable to change.
1b Particular about one aspect of their day only without being rigid about their overall routine (e.g., must have my coffee before driving the kids to school, I must have my evening run). It would significantly bother them if they had to change that aspect of their routine.
1c Provides a general overview of their routine but are bothered by disruption to routine or plans.
2 Provides a precise description of their routine or like to do certain things in a particular way and are bothered by disruption to their routine.

1 Would you describe yourself as easy-going, or as a bit particular or fussy?

What sort of things are you fussy about?

1A **Are you concerned about the way you look?**

What aspects of your appearance are you fussy about?

Would it bother you if you weren't able to _____ on a particular day?

How would it affect your daily activities or plans?

**Fussy: caring about details that no one else would notice (e.g. caring that there is a stray eyebrow hair or a tiny wrinkle on their sleeve).*

Scoring: __________

0 Not fussy. Doesn't care about details that no one else notices. Will not be bothered if things are not 'just so.'

1 Will say they are fussy, but the provided example will be something that others would notice (e.g. I like my shoes and belt to match). Feels bothered/disappointed if things aren't 'just so' but it will not affect daily life.

2 May show evidence of fussiness in interview (e. g. fixing their hair) and may appear as though they made an effort to look particularly groomed. Will report being fussy either in noticeable (e.g. shoes and belt) or unnoticeable domains (e.g. checking for eyebrow hair every morning; making sure that tie extends down to a certain point). Feels bothered if things aren't 'just so'. This may affect their daily activities.

1B ***Are you fussy about the way the cleaning gets done?***

Are you concerned about how cleaning is done or how something is cleaned?

What sort of things are you concerned about?

Why is that?

If someone else is doing the cleaning in a different way than you, would that ever bother you?

If someone is doing the cleaning in a different way than you, would you stop them?

Do you find that when others do the cleaning, they miss things that you would clean? What sorts of things?

* If the person is particular about people touching *their* things or room, rather than the cleaning itself, it's not a fastidious cleaning issue.

Scoring: __________

0 Does not have rigid approaches to cleaning. (e.g. "No problem as long as no food remains stuck on the plate")

1 An element of perfectionism is reported in cleaning – they like it to be done in a certain way and/or unease is felt when someone else is doing the cleaning in a different manner. They usually let others do the cleaning. They may overtake for reasons unrelated to a particular manner of cleaning (e.g. mother taking over when her child is cleaning because of poor results of the child's efforts)

2 Is a fastidious cleaner. Gives too much attention to detail that leads the participant to go beyond what is necessary for cleanliness. Unwilling to let someone else do the cleaning unless they do it "properly". Could do it as a hobby.

1C Are you particular about anything else?

Are you particular about how things are organized at home or at work?

Specific places? Size? Colour? Shape? Alphabetical order?

If you're doing something familiar, do you do it the same way?

For example, do you always take the same route to a certain destination? When making a familiar meal do you follow the steps in the exact same order?

Has anyone ever told you that things need to be done 'your way'?

Does it bother you when things aren't done your way?

Are you open to suggestions for new ways to do things?

Scoring: __________

0 No indication of rigidity. May prefer things to be done in a particular way, but is not bothered when things don't go their way. Is open to suggestions.

1 Prefers to do things in a particular way, and although it may bother them to do it a different way, they won't actively resist.

2 Participant is rigid and insistent on doing things his/her way. Others may have commented on their need to do things their way.

2 Do you have a hard time getting rid or things? (or throwing things away?)

Are there things that you have a hard time throwing out?

What sort of things are hard for you to throw out?

What would push you to get rid of those things?

Would you get rid of them if they were broken or no longer useful?

Would you get rid of them if they started taking up too much space?

Would you get rid of them if you moved houses?

Have other people ever told you that you should throw those out?

Did you throw them out after that?

Scoring: __________

0 No issues as long as the objects are not of monetary, practical, or sentimental value. May wait to decide if the object may be useful, but throws objects away once a decision about usefulness is made.

1 Participant has a tendency to keep things / some difficulty throwing things away but can easily throw things away if an incentive is given (e.g. no space in their home or instructed to do so by mother).

2 Participant keeps objects even if these are taking up valuable space. Others may have commented on their needing to throw things out.

3 **Do you think you are a perfectionist, and if so why?**

Does it sometimes take you a long time to accomplish a task because it has to be 'just right'?

Does anybody ever tell you that you are a perfectionist?

Sometimes perfectionism can cause problems for people. Does your perfectionism ever cause problems in certain areas of your life?

Social life? Work?

Does it lead to problems with time management?

Scoring: ___________

0 Doesn't identify self as a perfectionist, others do not identify person as perfectionist. Any perfectionistic tendencies will not have negative consequences.

1 Identifies self as a perfectionist. May spend extra time to accommodate for perfectionism, but there are no significant negative effects on functioning. Perfectionism remains generally a positive attribute for the individual.

2 Identifies self as a perfectionist. Others commenting on the perfectionism supports a high score. Perfectionism does interfere negatively with functioning (e.g. have a difficult time meeting deadlines, spend more time than required at work).

4 **How do you react when someone else makes a mistake?**

When someone makes a mistake do you point it out to them?

Example - You are at work and someone makes an error. How would you react?

Do you find it important to correct errors?

What sort of errors do you feel are important to correct?

My friend corrects people's grammar. Is there anything like that that you are a little bit picky about or that you correct, but others may not bother correcting?

Do you think that you correct errors that others wouldn't bother to correct?

Have people ever been offended when you corrected them?

Is that a personality trait of that person (i.e., is that person easily offended?)?

* *Look for compulsion to correct*

Scoring: ___________

0 Is not concerned by mistakes made unless these are important (e.g., if someone leaves the stove on).

1 Corrects critical and less critical errors, but makes a point of not to being an annoyance to others. May take mental note of the errors, but chooses not to point them out.

2 Finds it important to correct errors or have difficulty stopping themselves from doing so. Correcting errors may be more important to them than keeping the peace with others or they are aware that others are bothered by being corrected on less critical matters.

5 **Do you tend to worry?**

What sort of things do you worry about?
How often do you worry?
[Examples: Do you worry all the time? Just before a big event?]
Do you think that you worry a bit too much? Do others ting that you worry a bit too much?
Does your worry cause any problems in your life and if so what problems?
Does it cause problems with concentrating for example? Or with sleeping?

Scoring: __________

0 Worries about major life events (e.g. sick parent) or upcoming events (e.g. upcoming presentation). Worrying is infrequent and typically short-lived. Worrying does not interfere with daily activities.
1a Worries relatively frequently, but this does not affect daily functioning.
1b Worries rarely or only sometimes. Worrying interferes with daily activities such as focusing on work or sleeping only occasionally.
2 Worries often. Worrying interferes with daily activities or participant acknowledges that worrying is excessive or that other people find the worrying excessive.

6 Other than worries, is there any topic or idea you repeatedly think about?

Rewording - Are there any thoughts you repeatedly have?
How often to you think about it?
Do you think about it almost every week or almost every day?
Are these things you *worry* about? I mean things that often come into your mind, but that do not cause any worry.

Do you have a different way of looking at the world than other people or of seeing things in the world?

What is it?
Do you think like that often?
Do you notice things in your surrounding that other people don't tend to notice?
[Examples: Do you see patterns everywhere? Or see things in terms of numbers or shapes?]

Scoring: __________

0 Doesn't have recurrent non-distressing thoughts.
1 Thinks about a topic or sees things in a particular way sometimes. It isn't a circumscribed event (e.g. "If I play a computer game a lot, I feel the world looks a bit like the game and imagine I'm in the game." "If I am really involved in painting, when I look at things, I try to determine what colours make them up.")
2 Reports frequent recurrent thoughts. A topic is thought about often or they see the world in a special way most of the time (e.g. an artist almost always thinks about what colours make up the environment around him/her).

7 Are there certain aspects of your sensory environment (touch, taste, sight, sound, smell) that bother you?

Example - Do clothing labels or seams bother you? Certain textures of food?

Is there anything else you can think of that bothers you about the way things feel, taste, look or sound like?

Do you go out of your away to avoid these things?

Are there certain aspects of your sensory environment that you really enjoy?

Example - Do you find yourself running your hand across different surfaces? Do you like soft things?

Is there anything else you can think of that you particularly enjoy about the way things feel, taste, look, smell or sound like?

Scoring: __________

0 Does not have any unusual sensory interests or aversions.
1 May have a sensory interest or aversion, but does not avoid or seek out these experiences.
2 Has at least one clear sensory interest or aversion, and will avoid certain situations, or spend an extended amount of time engaging in a sensory interest.

Social Interactions

Now I am going to ask you a few questions about how you typically interact with other people.

1 How long does it take you to feel comfortable around someone new?

How long does it take you to feel comfortable around someone you like?
How many times do you have to meet someone before you feel comfortable with them?
Do you find that feeling comfortable is something that happens quite quickly or easily for you?
Are you usually happy when you are about to meet new people or do you feel more uncomfortable about it than happy?

Scoring: __________

0 Warms up to others quickly or at a rate judged to be typical. It can depend on the person they are warming up to. It should only take about a couple of meetings and not long at all to warm up.
1 Warms up a bit more slowly.
2 Takes a very long time to warm up to people. May not like the idea of meeting new people.

2 Do you usually prefer to spend time with others or to keep to yourself?

What makes you want to be alone?
Some people like to keep to themselves because they are a bit shy. Is that the case for you?
How often do you prefer to be on your own?
Are you able to give me a split of how much time you would like to spend on your own versus with others? For example, is it a 50/50 split?
Do you sometimes decide not to do activities with others because you prefer to be on your own? (e.g. If someone called you up for coffee when you were doing something on your own, would you generally accept the invitation and meet up with them?
Are you known for being a loner?
When you say time by yourself, does that mean time spent doing an activity without your partner or family?

If you are on your own would you prefer that others joined you or that they didn't?

How often would you prefer that others joined you?

Scoring: __________

0 Usually prefers to be around people rather than alone. Is rarely bothered by someone joining them.
1 Sometimes prefers to spend time alone. This is not due to an emotional reaction (e.g. wanting to be alone because they are frustrated vs. preferring to be alone when in a good mood). Makes a point of having their "alone time", but they rarely require this "alone time" Is bothered by someone joining them at this time.
2 Usually prefers to be alone because of lack of interest in others' company. Often feels bothered by others trying to join him/her. May not have many friends due to lack of interest in having friends (from poor interpersonal relationships item). May only enjoy the company of a few, select others (e.g. partner or best friend).

3 Most of us are awkward in social situations sometimes. Do you ever feel that you behave awkwardly in social situations?

Does it happen a lot?
Does it happen more than you think it happens to others?
Do you think other people notice when you are awkward or is it more of an internal feeling?
Is it sometimes a problem for your social life?
In what way is it a problem?

Scoring: ___________

0 Does not often behave awkwardly. Awkwardness does not impact their social life.
1a Perceive that they behave mildly awkwardly in social situations, a bit more so or more often than
1b others (may or may not be noticed by others). This does not negatively impact social functioning.
Behaves awkwardly occasionally. This does negatively impact on social functioning
2 Behaves awkwardly in social situations. This is noticed by others and/or is considered to negatively impact social functioning. May show awkwardness in the interview.

4 **Do you usually understand jokes that your friends make or that you see on TV?**
Do you sometimes not ‘get’ jokes?
Does it happen because you don’t understand the reference?
Is there a particular type of joke you have a harder time with? For example, some people find it difficult to understand sarcasm.
Do you think your sense of humour is different from others’?
Can you describe your sense of humour?
Do others find things funny that you don’t find funny? Are there things that you find funny that others don’t? What sorts of things?
Do other people notice that your sense of humour is different or that you have trouble understanding some jokes?
What TV show do you find funny?
**Note: Understanding of humour can be influenced by intellect and appreciation can be affected by culture.*

Scoring: __________

0 Nearly always understands jokes, unless you need specialized knowledge (e.g. physics jokes).
1 Has some difficulty understanding joke (e.g., they take a bit longer to ‘click’). This is not only because of unfamiliarity with references of jokes. Others are unlikely to notice their difficulty in understanding jokes.
2 Often doesn’t understand jokes or is particularly slow at understanding them. May be known as someone with trouble understanding jokes or requires explanations to understand jokes.

5 Do you sometimes make inappropriate comments?

Rewording - Do you sometimes say things that people find inappropriate?

Example - If somebody used the phrase 'that's going to cost you an arm and a leg' around an amputee.

Does it happen often or only rarely?

Do you feel that you make more inappropriate comments than other people?

When you make comments that you consider inappropriate, is it only with people you know well who wouldn't find it inappropriate? Or does it happen with people who would find it inappropriate as well

Does it affect you social life and if so how?

Does it sometimes interfere with how you get along with people?

**Inappropriate comments made intentionally in social settings where such comments are well received does not constitute inappropriate comments.* (marked with a leading asterisk)

Scoring: ___________

0 Doesn't generally make inappropriate comments.

1 They report a tendency to make inappropriate comments. This does not greatly impact on social functioning.

2 Has a tendency to make inappropriate comments. Others find the comment inappropriate. It Interferes with social functioning.

6 ***Do you sometimes have difficulty picking up on how someone else is feeling?***

Has anybody ever told you that you were not good at noticing how someone is feeling?
Does it ever happen that someone is upset and that you don't notice, but other people notice?
Does it happen rarely or often?
Does difficulty noticing how someone is feeling ever cause you problems in your social life?

Do you sometimes have a bit of a hard time seeing things from another person's point of view?

Can you imagine how another person might think of a situation in a different way than you?
Does that happen rarely or often?
Has it ever caused you problems in your social life?

Scoring: __________

0 No difficulty considering the point of view of another and "feels" for another.
1a Doesn't easily consider another's point of view, but can generally pick up on their emotions.
1b Easily considers another's point of view, but has difficulty picking up on how someone is feeling.
2 Has difficulty considering another's point of view and doesn't easily pick up on another's emotions.

7 ***When someone who is close to you is feeling a certain way, do you tend to experience a similar feeling?***

So for example, if your partner/close friend is upset, does it upset you to see them this way?
Is this true in most situations? Or for only extreme emotions/situations?

How do you feel when you find out that you've hurt someone's feelings or upset them?

What do you do in those situations?
Do you try to patch things up?
What is the aim?

Scoring: __________

0 Shares emotions and "feels" for another. Feels bad when they upset someone, and will try to patch things up with another person so that the other person will feel better, not just themselves.
1 Doesn't generally share emotions with others, but feels bad when they know that they've upset someone and will make an effort to fix things.
2 Does not "feel" for another or share their emotions and/or does not feel bad if they upset someone. May attempt to patch things up, but out of obligation, rather than a desire to make the other person feel better.

8 Are you easily or frequently frustrated?

Does it take a lot to make you frustrated or angry?

Can you give me an example of what would make you frustrated?

Are there daily things that often make you frustrated?

How do you show your frustration?

What do you do that makes people know you are frustrated?

Do you ever take out your frustration on other people or on objects?

Example - By being short tempered or snappy with them for example?

**Aggression: Taking it out on others (people or objects) by screaming, physically hurting self or others, making hurtful comments, or behaving in a passive-aggressive manner.*

Scoring: ___________

0 Does not easily get frustrated and when they are frustrated they do not act aggressively.

1a Is not easily frustrated but when their threshold is reached they react aggressively (e.g. when they miss their plane, they threaten the boarding staff).

1b Has a low tolerance to frustration but does not react aggressively (e.g. become frustrated in daily traffic).

2 Has a low tolerance for frustration and acts out aggressively.

9 **Are you someone who likes to share their opinion?**
Do you share your opinion rarely or often?
Are you a bit stubborn with your opinions?
Rarely or often?
Do you or others think that you are opinionated?
When do you choose to hold back your opinions?
If others don't share your opinion will you share it?
If it may offend others, will you voice your opinion?
Do you feel that your opinion is better than that of others?
*Specify environment (eg academic setting requires voicing and sticking strongly to opinion).

Scoring: __________

0 Does not express that he/she likes to share their opinions, but is not necessarily uncomfortable with it. Will consider the feelings of others when sharing their opinion and will back off if the situation becomes uncomfortable.
1 Is Likes to share their opinions, but will typically hold back if their opinion would upset anyone or might be viewed as inappropriate. Is rarely stubborn about their opinion and is not necessarily known for being opinionated.
2 Will voice their opinion even in inappropriate situations and regardless of the considerations listed in 0 (not necessarily always). They like or find it important to express their opinions. They are stubborn with their opinion.
**Inappropriate: offends someone or is situationally inappropriate*

10 **How do you compare yourself to other people?**
Do you sometimes consider yourself better than other people?
Can you give me an example of who you might feel superior to?
Do you consider yourself better than some people in some specific areas?

Scoring: __________

0 Has no feelings of superiority to others. If they state they are superior in a few domains (e.g. Are a better cook than some people they know), they are *reluctant to describe themselves in those terms.*
1 Feels superior to some others (e.g. I'm better than many people in prison). May be reluctant to describe themselves in those terms and/or are not reluctant to mention they are better than others in some aspects of life.
2 Is not reluctant to state that they are better than others. May take the opportunity to talk about themselves in grandiose terms.

11 **When you are with your family or friends, are you affectionate? How do you show it?**

[Example] Do you like to give hugs for example?
Do you show them affection often or do you tend to be more reserved?

[For those who are not keen on affection]
The people who know you well, do they think you are not particularly affectionate?

How do you react when someone tries to give you a hug for example?
Have you ever gone out of your way to avoid receiving affection from someone?
Would you say that you are less likely to be the one to go forward and give the affection, but if someone gives you affection, you are comfortable?
Does it bother you if you have to cancel an activity or replace it with another one?

**Appropriate affection: occasionally cuddling grandma, increased affection with partner vs others, etc.*
Inappropriate: Overly affectionate in an inappropriate context.
** Affection = physical expression of fondness*

Scoring: ___________

0 Does like expressing/receiving affection. Often displays their affection.
1 Does not always like expressing or receiving affection, but has not taken steps to avoid affection.
2 Does not like expressing/receiving affection. They are known for being non-affectionate. They have actively tried to avoid affection (e.g., busying self in kitchen so that they do not have to greet arriving guests) or are very uncomfortable when receiving affection.

12 **How many close friends do you have?**

Are these people you are able to talk to about personal matters and go to for help?

Think about your closest friend. What makes you close?

Do you talk to them often?

Do you go to them when you need comfort or support?

Are you someone who makes close friends easily?

What makes it difficult for you?

Do you find it a bit challenging to maintain a close friendship?

Do you find it a bit of a chore to keep in touch with friends?

[If suspecting aloofness]

Are you interested in having more friends or closer friendships?

[Rewording] Are you happy/content with the number of friends you have?

**Close friendship: sharing, confiding, helping, trusting, can be yourself around the person, etc.*

** Check social circumstances (eg. typically have many friends but have recently moved).*

Scoring: ___________

0 Has close friends with no difficulty forming or maintaining close friendships.

1a Has a number of close friends but has some difficulty forming or maintaining close friendships.

1b Has few close friends (one or two), but no trouble maintaining the relationships.

2 Has one to two or no close friends and finds it difficult to form close friendships or maintain them.
