# Supplementary material for: Tracing Autism Traits in Large Multiplex Families to Identify Endophenotypes of the Broader Autism Phenotype
Source: Int J Mol Sci. 2020 Oct 27;21(21):7965. doi: 10.3390/ijms21217965 (PMC7663259; doi:10.3390/ijms21217965)
Supplement: Supplementary file 1 [file ijms-21-07965-s001.zip › Supplementary_IJMS_v2.docx]

# Supplementary Data

Supplementary Table S1. Identification of BAP traits through expansion of the BAP domains

| BAP domains | Expanded traits |
| --- | --- |
| Communication  (speech and literacy) | Reduced capacity for clear narrative  Difficulty answering open ended questions  Reduced quantity of verbal output  Speech has little variation in tone (i.e. monotonous)  Unusual speech volume  Precise articulation and language  Use of an accent* |
| Social communication  (pragmatics and relationships) | An unusual or awkward greeting style  A limited capacity to develop rapport with assessors  Unusual eye gaze  Awkward social interactions  Making inappropriate or awkward comments either on history or during assessments  Tangential pragmatic style  Terse pragmatic style  Tendency to monologue rather than participate in reciprocal conversation  Opinionated in conversation  Overly technical language  Little appreciation of humour (during the Cartoon task)  Inflexible to intentional errors*  Tendency to anger easily  Narcissistic personality style  Self perception incongruent with views of others  Aloof personality style  Difficult or limited interpersonal relationships  Reduced affection  Reduced emotional empathy  Reduced cognitive empathy  Excessive worry |
| Circumscribed interests | Preference for structure in activities of daily living  Fastidious regarding personal appearance  Fastidious cleaning  Hobby or interest of unusual intensity, or restricted range of interests relative to peers  Large collections or hoarding of items  Focus on technicalities or minutiae  Recurrent thoughts (distressing)*  Recurrent thoughts (not distressing) |
| * BAP trait was removed from the final list (see text for details). | |

Supplementary Table S2. Gottesman and Gould criteria for an endophenotype.

| 1. Segregate with illness in the general population 2. Be heritable 3. Be state independent, manifesting whether illness is present or not. 4. Cosegregate with the disorder within families. 5. Be present at higher rates within affected families than in the general population. 6. Be characteristics that can be measured reliably, and specific to the illness of interest. |
| --- |

*Note. Adapted from Gottesman and Gould (2003) and Beauchaine and Theodore (2017)*


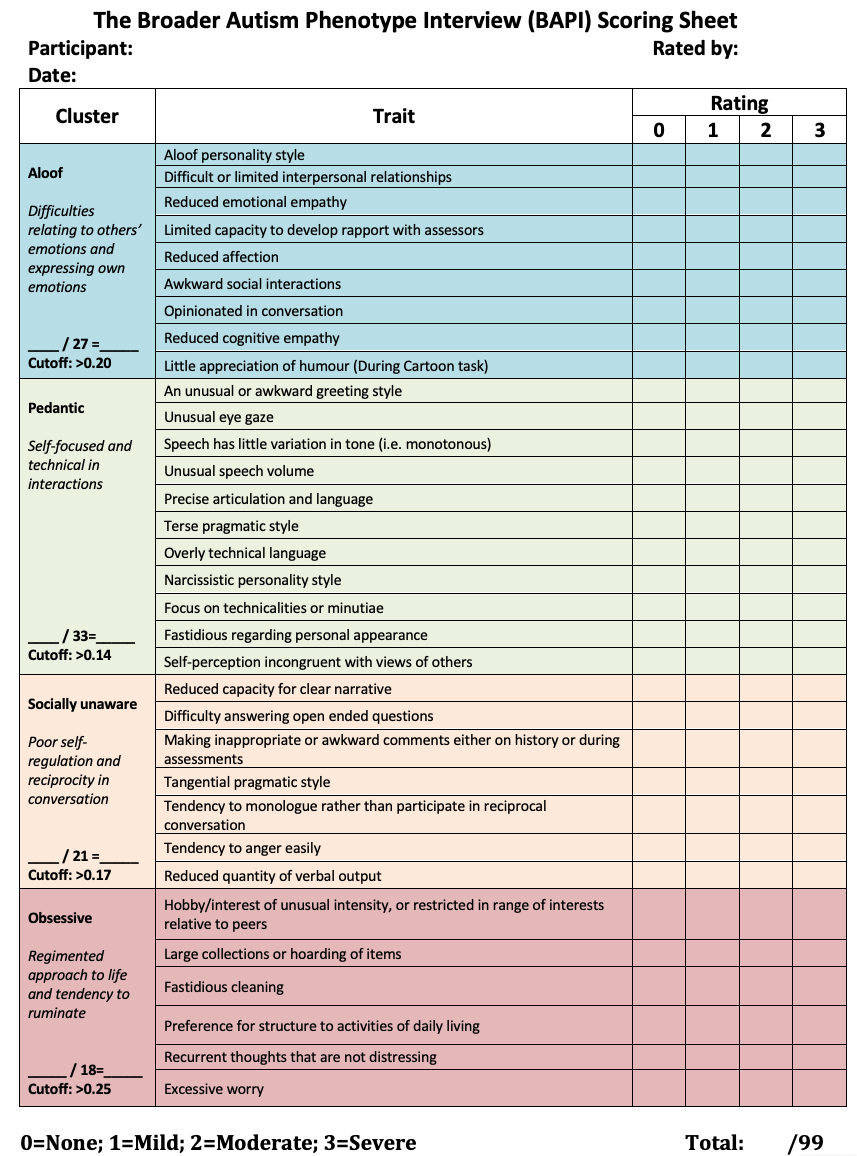


**Supplementary Figure S1.** Endophenotype scoring from clinical ratings of 33 traits, based on information gathered from the Broader Autism Phenotype Interview (See Supplementary materials)
